# The effect of periodic resistance training on obese patients with type 2 diabetic nephropathy

**DOI:** 10.1038/s41598-024-53333-4

**Published:** 2024-02-02

**Authors:** Sumei Li, Shouping Yuan, Jintian Zhang, Feipeng Xu, Feng Zhu

**Affiliations:** 1 Department of Endocrinology, Teaching Hospital, The First Hospital of Putian City, Fujian Medical University, Putian, Fujian China; 2 Department of Pathology, Putian University, Medical University, Putian, Fujian China

**Keywords:** Endocrinology, Nephrology

## Abstract

Resistance training is an exercise against resistance designed to train the endurance and strength of muscle. To observe the effect of intervention of periodic resistance training on obese patients with type 2 diabetic nephropathy. A total of 60 obese patients with type 2 diabetic nephropathy were randomized into resistance training group and aerobic exercise group (30 patients each group) for observing the changes of blood glucose, body weight, blood lipid, insulin resistance, serum creatinine (Scr), urinary microalbumin, urinary albumin excretion rate (UAER) calculated by urinary creatinine, and glomerular filtration rate (GFR) after 12 weeks of intervention, and relevant significance as well. The number of patients with hypoglycemia during the intervention was also recorded. After 12 weeks of intervention, the weight, Body mass index (BMI), Waist, Triglyceride (TG), Cholesterol (TC), Low-density lipoprotein cholesterol (LDL), Fasting glucose (FBG), Fasting insulin (FINS), Glycosylated hemoglobin (HbA1c) and urine Albumin–Creatinine Ratio (uACR) were decreased and GFR was increased in both groups (P < 0.05), but the effect was more significant in the resistance training group. GFR was increased from 92.21 ± 10.67 mL/(min·1.73 m^2^) to 100.13 ± 12.99 mL/(min·1.73 m^2^) in resistance training group (P < 0.05). In the aerobic exercise group, GFR was increased from 89.98 ± 9.48 mL/(min·1.73 m^2^) to 92.51 ± 11.35 mL/(min·1.73 m^2^) (P > 0.05). Periodic resistance training can not only control the weight, blood sugar and blood lipid of obese patients with type 2 diabetic nephropathy, but also improve the urinary albumin excretion rate and glomerular filtration rate of early obese patients with type 2 diabetic nephropathy, and delay the progression of diabetic nephropathy. It is an effective non-drug intervention.

## Introduction

As a global disease, diabetes has affected about 463 million people aged 20–79 worldwide with a prevalence rate of about 9.3%. According to an estimation, in 2025, there will be about 700 million patients with diabetes, and the prevalence rate will be increase by 10.9%^1^. The incidence rate of diabetic nephropathy in patients with type 2 diabetes (T2DM) is about 40% at present, and is expected to increase to 50% by 2025^2–4^. The number of obesity cases is also on the rise. The higher the body mass index, the higher the risk of type 2 diabetes. For the resistance training, an exercise against resistance designed to train the endurance and strength of muscle, this study adopted a resistance band. The band is cheap and portable, the requirements for training site are relatively low, and the compliance of subjects is high.

Globally, scholars have found that the exercise against resistance is beneficial to weight loss and blood glucose control in recent years. However, the application of resistance training has not been popularized among obese patients with diabetic nephropathy, and so, relevant data are to be provided. This study was to observe the therapeutic effect of periodic resistance training on obese patients with type 2 diabetic nephropathy.

## Methods

### Study design

The subjects were randomized into two groups, namely resistance training group and aerobic exercise group. Before test, all of the subjects were provided with relevant education, and given diet instructions. Regular diabetic diet control was given with 30–35 kcal of energy per kilogram of ideal body weight per day. Ideal weight (kg) = height (cm) − 105. Their original medication regimens were maintained during test. For the control group, the patients performed aerobic exercise. Specifically, they warmed up for 5 min before exercise, ran slowly for 30 min, and rested for 5 min after exercise, that is, the exercise lasted about 40 min each time, and 5 times a week. For the observation group, a portable resistance band was used for movements as follows after 5 min of warm-up by walking slowly: squat with resistance band, and perform back neck flexion and extension with resistance band, 15–20 times each movement for females and 20–25 times each movement for males. Complete 3 sets of movements with a 2–3 min interval between sets. The exercise lasted about 40 min each time, with target heart rate (60–80% of the maximum heart rate) maintained for 20–40 min, and 5 times if exercise were performed every week. All of the subjects were followed up by telephone every month for recording their exercise, blood glucose test and drug use. For the included subjects, baseline data at inclusion as well as data 12 weeks after intervention were evaluated. The patients’ height, waist circumference and body weight were monitored for body mass index (BMI) calculation, and the FBG and FINS were monitored for calculating Homeostasis Model Assessment-Insulin Resistance (HOMA-IR) as follows: HOMA-IR = FBG·FINS/22.5. At the same time, HbA1c, TC, LDL-C, HDL-C, TG, Scr, uACR and GFR were tested. GFR = (186 × Scr) − (1.154 × age) − 0.203 × (0.742 if female). Scr is serum creatinine (mg/dL), and the unit conversion of blood creatinine is 1 mg/dL = 88.41 μmol/L. For all of the subjects, the person-times of hypoglycemia during the 12-week intervention were recorded. Symptoms of hypoglycemia are as follows: hunger, cold sweat, palpitation, hand shaking, fatigue, and etc. A hypoglycemic event occurs if blood glucose < 3.9 mmol/L.

### Participants

A total of 60 obese patients with type 2 diabetic nephropathy treated at the Endocrinology Clinic of our hospital between June 2021 and December 2022 were included. The patients were randomized into resistance training group and aerobic exercise group (30 cases each group) for observing the changes of relevant indicators after 12 weeks of intervention, and relevant significance as well. Inclusion criteria: Patients aged 20–50 years old with BMI ≥ 28 kg/m^2^, HbAlc 7–9%, and diagnosed as T2DM with symptoms consistent with the 1999 WHO diagnostic criteria for diabetes with disease course ≥ 1 year. Diagnosis criteria for early-stage diabetic nephropathy: increased urinary albumin excretion rate. In this study, urinary albumin/urinary creatinine ratio was used to express urinary albumin excretion rate, 30 μg/mg < urine albumin–creatinine ratio (uACR) < 299 μg/mg, and the results of both tests were abnormal. Glomerular filtration rate (eGFR) was > 60 mL/(min·1.73 m^2^). Other possible causes of urinary microalbumin increase were excluded. Exclusion criteria: (1) Patients with serious infections, and serious hyperglycemia for diabetics (e.g., ketoacidosis, lactic acidosis, hypertonic state, and etc.); (2) Patients with autoimmune diseases; and (3) Patients with serious dysfunctions or insufficiencies of vital organs like the heart, liver, kidney and brain. 5 people in the resistance training group and 1 in the aerobic exercise group quit the experiment. In the resistance training group, there were 11 males and 14 females. In the aerobic exercise group, there were 13 males and 16 females. Neither group received ACEI or ARB or SGLT2, drugs that may affect urinary protein excretion, during the study period.

### Statistical analysis

SPSS 22.0 software was used for a statistical analysis. The results were expressed as x ± s, and the non-normal distribution was expressed as median and inter-quartile range. The inter-group and intra-group comparisons of relevant data were carried out by t test or rank sum test, and if P < 0.05, there was statistical significance.

### Ethics approval and consent to participate

Ethical clearance and approval were obtained from Ethics Committee of the First Hospital of Putian, Fujian , China. All study participants were informed about the purpose of the study and additional information was given as they need. Written informed consent was obtained from all participants. We had complied with the Declaration of Helsinki Ethical Principles for medical research involving human subjects.

## Results

### Baseline characteristics

There was no statistical significance in gender, age, disease course, weight, BMI, waist, TG, TC, LDL, HDL, FBG, FINS, HbA1c, Scr, UAER or GFR before intervention (P > 0.05), as shown in Table 1.

**Table 1:** Baseline characteristics of patients ($\overline{x} \pm s$).

Project	Resistance training group	Aerobic exercise group	P
Age (years)	36.60 ± 13.16	37.01 ± 13.02	0.649
Waist (cm)	108.62 ± 12.04	107.59 ± 12.21	0.708
Weight (kg)	78.29 ± 15.22	77.45 ± 14.55	0.823
HbA1C (%)	8.42 ± 1.51	8.39 ± 1.52	0.625
BMI (kg/m^2^)	29.98 ± 5.77	29.93 ± 6.01	0.894
FINS (pmol/L)	48.65 ± 17.53	47.91 ± 14.14	0.677
FBG (mmol/L)	9.08 ± 2.69	8.98 ± 2.56	0.945
TG (mmol/L)	1.81 ± 0.52	1.88 ± 0.73	0.754
HOMA-IR	5.91 ± 2.69	5.84 ± 2.71	0.691
TC (mmol/L)	4.56 ± 0.61	4.68 ± 0.65	0.731
HDL (mmol/L)	1.01 ± 0.19	1.08 ± 0.33	0.579
LDL (mmol/L)	2.79 ± 0.65	2.76 ± 0.68	0.833
Scr(μmol/L)	81.23 ± 18.35	82.00 ± 19.31	0.669
uACR (μg/mg)	96.46 ± 20.76	92.15 ± 18.85	0.846
GFR(mL/(min·1.73 m^2^)	92.21 ± 10.67	89.98 ± 9.48	0.549

After 12 weeks, the weight, BMI, waist, TC, TG, HDL,LDL, FBG, FINS and HbA1c were all decreased for both groups (P < 0.05). HDL was increased in the two groups, but the difference was not statistically significant (P > 0.05). The body mass, blood lipid and blood glucose in the resistance training group were significantly improved as compared with those in the aerobic exercise group (P < 0.05). See Table 2.

**Table 2:** Changes of indicators before and after intervention of different exercise patterns in the two groups ($\overline{x} \pm s$).

Project	Resistance training group (n = 25)		P	Aerobic exercise group (n = 29)		P
	Before the intervention	After the intervention		Before the intervention	After the intervention	
Waist (cm)	108.62 ± 12.04	99.02 ± 13.22	0.000	107.59 ± 12.21	103.45 ± 10.62	0.000
Weight (kg)	78.29 ± 15.22	73.44 ± 14.32	0.000	77.45 ± 14.55	75.31 ± 12.86	0.000
BMI (kg/m^2^)	29.98 ± 5.77	27.52 ± 5.64	0.000	29.93 ± 6.01	28.16 ± 5.73	0.000
HbA1C (%)	8.42 ± 1.51	7.63 ± 1.51	0.000	8.39 ± 1.52	8.07 ± 1.46	0.000
FBG (mmol/L)	9.08 ± 2.69	7.92 ± 1.48	0.000	8.98 ± 2.56	8.29 ± 2.24	0.000
FINS (pmol/L)	48.65 ± 17.53	23.38 ± 9.13	0.000	47.91 ± 14.14	27.33 ± 10.65	0.000
HOMA-IR	5.91 ± 2.69	2.45 ± 1.11	0.000	5.84 ± 2.71	2.77 ± 1.21	0.000
LDL (mmol/L)	2.79 ± 0.65	2.25 ± 0.41	0.000	2.76 ± 0.68	2.71 ± 0.48	0.000
HDL (mmol/L)	1.01 ± 0.19	1.15 ± 0.33	0.136	1.08 ± 0.33	1.20 ± 0.28	0.201
TG (mmol/L)	1.81 ± 0.52	1.14 ± 0.20	0.000	1.88 ± 0.73	1.49 ± 0.41	0.000
TC (mmol/L)	4.56 ± 0.61	4.03 ± 0.44	0.000	4.68 ± 0.65	4.25 ± 0.37	0.000
Scr (μmol/L)	81.23 ± 18.35	72.52 ± 16.41	0.124	82.00 ± 19.31	76.23 ± 15.89	0.223
uACR (μg/mg)	96.46 ± 20.76	77.15 ± 17.58	0.000	92.15 ± 18.85	84.56 ± 17.49	0.000
GFR (mL/(min·1.73 m^2^)	92.21 ± 10.67	100.13 ± 12.99	0.000	89.98 ± 9.48	92.51 ± 11.35	0.312

### Comparison of renal function indicators for the two groups after treatment

The serum creatinine level was decreased after treatment for both groups, but there was no statistical significance (P > 0.05). The UARE level was reduced after exercise with statistical significance (P < 0.05) for both groups, but the reduction rate in the resistance exercise group higher than in the aerobic exercise group (P < 0.05). GFR was increased after resistance training with statistical significance (P < 0.05), while there was no significant change after treatment in the Table 2.

**Figure 1:**
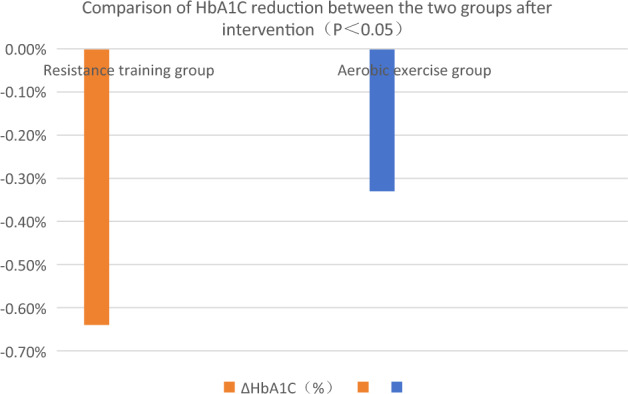
Comparison of HbA1C reduction between the two groups after intervention (P < 0.05).

**Figure 2:**
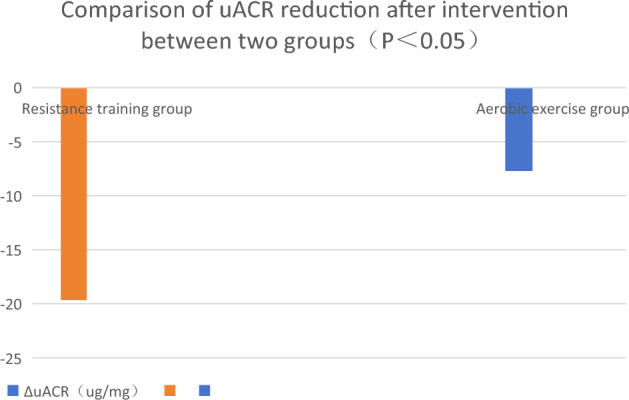
Comparison of uACR reduction after intervention between two groups (P < 0.05).

**Figure 3:**
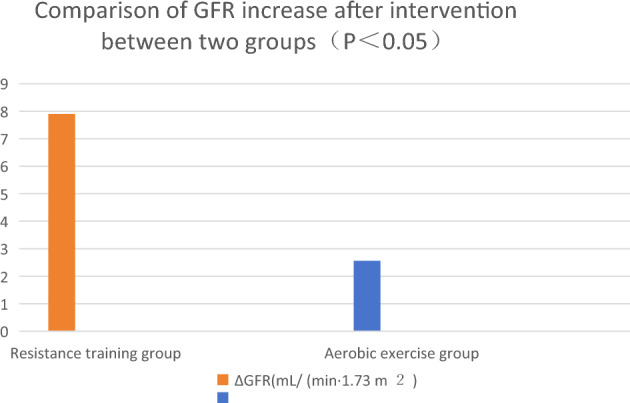
Comparison of GFR increase after intervention between two groups (P < 0.05).

Comparison of the differences in the outcomes of each intervention (ΔHbA1c, ΔuACR and ΔGFR): ΔHbA1c decreased by 0.64% and 0.33% in resistance training and aerobic exercise groups, respectively (P < 0.05). ΔuACR decreased by 19.67 μg/mg and 7.65 μg/mg in resistance training group and aerobic exercise group, respectively. ΔGFR increased by 7.90 mL/(min·1.73 m^2^) and 2.56 mL/(min·1.73 m^2^) in resistance training group and aerobic exercise group, respectively. See Figs. 1, 2 and 3.

### Hypoglycemia in weeks 3–4 of intervention

A total of 8 person-times of hypoglycemia occurred in the resistance training group, that is, 2 person-times of peripheral blood glucose < 3.9 mmol/L. In the aerobic exercise group, and 2 person-times of hypoglycemia occurred in the aerobic exercise group, that is, 0 person-time of peripheral blood glucose < 3.9 mmol/L. Hypoglycemic drugs were appropriately adjusted after hypoglycemia occurred, and no hypoglycemic event occurred again in weeks 5–12 of intervention for both groups.

### Follow up for willingness to adhere

After the study, a questionnaire survey was conducted on the willingness to continue resistance training mode. The results showed that the resistance training group had lower long-term adherence than the aerobic exercise group. Most patients think that the exercise load of resistance training is strong, and the short-term phase can adhere to it, but the long-term persistence worries that with the growth of age, the body can not tolerate. The results are shown in Table 3.

**Table 3:** Differences in intentions after completion of projects between the two groups.

	Resistance training group	Aerobic exercise group	p
Number of people who cannot stick to it (percentage)	5 (16.7%)	1 (3.3%)	< 0.05
Number of People willing to stick with it in the short term (proportion)	15 (50%)	28 (93.3%)	< 0.05
Number of people willing to stick with it for the long term (proportion)	8 (26.7%)	24 (80%)	< 0.05

## Discussion

In this study, obese patients with type 2 diabetic nephropathy were given 12 weeks of resistance training or aerobic exercise in addition to drugs for blood glucose control. After intervention, the weight, BMI, Waist, TG, TC, LDL, FBG, FINS, HbA1c and uACR were decreased and GFR was increased in both groups (P < 0.05), but the effect was more significant in the resistance training group. GFR was increased from 92.21 ± 10.67 mL/(min·1.73 m^2^) to 100.13 ± 12.99 mL/(min·1.73 m^2^) in resistance training group (P < 0.05). In the aerobic exercise group, GFR was increased from 89.98 ± 9.48 mL/(min·1.73 m^2^) to 92.51 ± 11.35 mL/(min·1.73 m^2^) (P > 0.05).

The incidence of type 2 diabetes is increasing year by year. The main environmental factors of type 2 diabetes include excessive intake of high-calorie diet, low consumption due to insufficient physical activity, obesity and so on. Worldwide, the prevalence of obesity has increased the morbidity and mortality of cardiovascular and cerebrovascular diseases, diabetes, nephropathy, tumors and etc.^5^. A relevant study has shown that increased daily exercise and dietary intervention can reduce the incidence of type 2 diabetes, and lower the occurrence and development of diabetes microvascular-related organ complications as realizing a weight loss effectively^6^. As a common microvascular complication of diabetes, diabetic kidney damage may affect the patients’ quality of life seriously during its occurrence and development. Albuminuria monitoring and GFR estimation should be carried out among diabetic patients every year^7^. Chronic kidney disease (CKD) is defined as kidney damage resulting from reduced GFR or increased albumin excretion. However, exercise training is an important non-pharmacological intervention to improve and prevent diabetes and CKD^8^.

This study found that the reduction in HbA1C and uACR was greater in the resistance training group than in the aerobic exercise group, and the increase in GFR was also greater than in the control group. It shows that the increase of GFR and the decrease of UAER after resistance training intervention may be due to the direct benefit of resistance training on the one hand, but may be due to the increase of GFR and the decrease of UAER caused by better control of blood glucose and weight after resistance training on the other hand. Deus et al. also pointed out that, Resistance training can effectively control glucose and blood lipid in kidney disease. Resistance training-induced glucose regulation prevents the progression of CKD^9^. Under long-term aerobic exercise training, the body can obtain more mitochondria and higher capillary density, increase the maximum oxygen consumption and improve the secretion and sensitivity of insulin gradually, thus lowering the blood glucose^10–12^. Mostly, the staged resistance training promotes muscle synthesis as well as glucose uptake through enhancing the oxidative capacity of mitochondria. After the enhanced exercise, the synthesis of muscle glycogen can enhance the synthesis of insulin related positive regulators to further reduce the insulin resistance of skeletal muscle, regulate the body's glucose metabolism, promote its glucose uptake, and increase the glucose clearance rate, thus reducing HbA1c^13,14^. There has been a definite consensus on the effect of aerobic exercise on improvement of diabetes, but there is still no sufficient data supporting the effect of resistance training in obese patients with type 2 diabetic nephropathy. For this, this study was conducted.

In this study, both waist circumference and body mass decreased, the blood glucose was controlled, and the insulin resistance was alleviated. Overall, relevant lipid metabolism indicators were improved for the subjects. Skeletal muscle is critical for body function and metabolic health. As one of the most effective non-pharmacological external variables, resistance training can stimulate factors associated with muscle hypertrophy, and thus reduce muscle loss^15^. However, some other studies have suggested that resistance training has more important effects on body composition, muscle strength, and metabolic risk factors^16^. Marques EA et al. reported changes of metabolic parameters in 60 obese women after 20 weeks of resistance training without diet control, and as a result, the resistance training reduced the Tc and TG levels in the obese women significantly^17^. In a study of Amouzad^18^, after 8 weeks of resistance training in patients with type 2 diabetes, their blood glucose, insulin, and insulin resistance index were significantly lowered as compared with the control group. A meta-analysis has shown that resistance training may improve the blood glucose as well as the strength and endurance of muscle in elderly patients with T2DM^19^.

Similar to other studies, this study showed that the blood glucose, blood lipid and body weight of patients in the resistance training group decreased significantly as compared with those in the aerobic exercise group. Resistance training is an effective strategy for lowering HbA1c for type 2 diabetes^20^. Ranasinghe proposed that the high baseline HbAlc (> 7.5%) group had a more significant effect (resistance training > aerobic exercise)^21^. In terms of hypoglycemia, the frequency of hypoglycemia was higher in the resistance training group at the initial stage of the study, but did not occur any more after adjusting the glucose-lowering regimen. Therefore, it was considered relevant to the effect in lowering blood glucose more significantly.

This study showed that in patients at an early stage of T2DM nephropathy, 12 weeks of resistance training significantly reduced UAER, an renal function index, and increased GFR, and the effect was more obvious than that in the control group, suggesting that resistance training may improve the renal function in patients with T2DM. A 1% reduction in HbAlc was associated with a 37% reduction in microangiopathy risk in patients with type 2 diabetes^22^. In patients with diabetic nephropathy, proper physical exercise may reduce the albuminuria and alleviate the progression of nephropathy^23,24^. Some animal experiments have shown that resistance training can enhance insulin-induced vasodilation function in rats^25^. The function of vascular endothelium was improved by increasing the release of nitric oxide and the level of nitric oxide synthetase, and different intensity and frequency of resistance training improved the vascular endothelium to different degrees^26^. Resistance training may further affect the microvascular function in T2DM patients through improving their micro-vasodilation and insulin signaling^27^. Zaki et al. found that the exercise training mode of aerobic exercise and resistance exercise at the same time can effectively improve the metabolic markers, blood lipids and body composition of T2DM patients^28^. In this study, the improvement of renal function in T2DM patients may be related to resistance training.

This study has several limitations. The number of cases in this study is limited, and the follow-up time is only 12 weeks, so longer follow-up observation and more cases are needed for the later improvement data of diabetic nephropathy. Subsequently, we are also observing the effects of resistance training combined with drugs (ACEI, ARB, SGLT2, etc.) or combined aerobic exercise in early diabetic nephropathy patients.

## Conclusions

Periodic resistance training can not only control the weight, blood sugar and blood lipid of obese patients with type 2 diabetic nephropathy, but also improve the urinary albumin excretion rate and glomerular filtration rate of early obese patients with type 2 diabetic nephropathy, and delay the progression of diabetic nephropathy. It is an effective non-drug intervention.

## Data Availability

The datasets used in the analyses described in this study are available from the corresponding author on reasonable request.

## Abbreviations

T2DM: Type 2 diabetes
FBG: Fasting glucose
FINS: Fasting insulin
BMI: Body mass index
HbA1c: Glycosylated hemoglobin
TC: Cholesterol
LDL-C: Low-density lipoprotein cholesterol
HDL-C: High-density lipoprotein cholesterol
TG: Triglyceride
Scr: Serum creatinine
UAER: Urinary albumin excretion rate
uACR: Urine albumin–creatinine ratio
GFR: Glomerular filtration rate
CKD: Chronic kidney disease
